# Randomized, Double-Blind, Placebo- and Positive-Controlled Crossover Study of the Effects of Tebipenem Pivoxil Hydrobromide on QT/QTc Intervals in Healthy Subjects

**DOI:** 10.1128/AAC.00145-21

**Published:** 2021-06-17

**Authors:** Vipul K. Gupta, Gary Maier, Paul Eckburg, Lisa Morelli, Yang Lei, Akash Jain, Erika Manyak, David Melnick

**Affiliations:** a Spero Therapeutics, Inc., Cambridge, Massachusetts, USA; b Maier Metrics and Associates, LLC, Falmouth, Maine, USA; c Ribon Therapeutics, Inc., Cambridge, Massachusetts, USA

**Keywords:** QT interval, pharmacokinetics, tebipenem pivoxil hydrobromide

## Abstract

Tebipenem pivoxil hydrobromide (TBP-PI-HBr) is an orally available prodrug of tebipenem (TBP), a carbapenem with *in vitro* activity against multidrug-resistant Gram-negative pathogens. This study evaluated the effects of single therapeutic and supratherapeutic doses of TBP-PI-HBr on the heart rate-corrected QT interval (QTc) by assessing the concentration-QT interval relationship using exposure-response modeling. This was a randomized, double-blind, placebo- and active-controlled, single-dose, four-way crossover study. Subjects received single oral doses of TBP-PI-HBr at 600 and 1,200 mg, placebo, and positive control (moxifloxacin at 400 mg). Cardiodynamic electrocardiograms (ECGs) and blood samples were collected in each period. Twenty-four subjects were enrolled. TBP-PI-HBr had no clinically significant adverse effects on heart rate or ECG parameters. The model-predicted slope suggests that the baseline-corrected difference in heart rate from placebo was not importantly affected by plasma TBP concentrations, supporting the use of the QT interval corrected by Fridericia’s method as an appropriate correction. The model-predicted difference in QTc at the mean maximum concentration (*C*_max_) for TBP had negative predicted values for each dose, and no QTc prolongation was detected following TBP-PI-HBr at 600 mg or 1,200 mg. Assay sensitivity was established with moxifloxacin at 400 mg. Exposure to TBP increased in a dose-dependent manner with 600- and 1,200-mg doses. The TBP area under the concentration-time curve from time zero to infinity and *C*_max_ with the 1,200-mg dose were 1.8- and 1.3-fold greater, respectively, than those with the 600-mg dose. TBP-PI-HBr was generally safe and well tolerated, with no effect in QT interval prolongation.

## INTRODUCTION

Drug-induced prolongation of the QT interval has the potential to cause fatal ventricular arrhythmias (1). A number of antimicrobials, particularly quinolone antibiotics, are associated with a small but clinically significantly increased risk of QT interval prolongation (2–5). Among carbapenems, imipenem-cilastatin is reported to be associated with a severalfold increase in the risk for QT interval prolongation (5); however, cardiovascular effects or prolonged QT intervals have not been reported as an increased risk with other carbapenems. Nevertheless, most new chemical entities undergo rigorous evaluation of QT interval prolongation to assess the potential to delay cardiac repolarization, which leads to the development of ventricular arrhythmias (i.e., torsade de pointes) and may result in sudden cardiac death (6, 7).

Tebipenem pivoxil hydrobromide (TBP-PI-HBr) (formerly SPR994) is an orally available prodrug of tebipenem (TBP), a carbapenem with *in vitro* activity against multidrug-resistant (MDR) Gram-negative pathogens, including quinolone-resistant and extended-spectrum β-lactamase (ESBL)-producing *Enterobacteriaceae* (8–12). Time-dependent pharmacokinetic (PK)/pharmacodynamic parameters (free drug area under the concentration-time curve [*f*AUC]/MIC · 1/tau, where tau represents the length of the dosing interval) are the best predictors of the antimicrobial activity of TBP (13, 14). Previously, the PK of TBP were evaluated in a single-dose and multiple-ascending-dose study in healthy subjects (15). The PK profile of TBP generally was dose proportional and linear after single doses of 100 to 900 mg. After multiple daily doses of 300 and 600 mg every 8 h, TBP demonstrated dose-proportional PK with no accumulation over 14 days (15).

The objective of this study was to evaluate the effects of single therapeutic (600 mg) and supratherapeutic (1,200 mg) doses of TBP-PI-HBr on the heart rate (HR)-corrected QT interval (QTc) by assessing the concentration-QT interval (C-QT) relationship using exposure-response modeling.

## RESULTS

### Subjects.

Twenty-four subjects were randomly assigned to study treatments; all 24 subjects completed the study and were included in analyses of cardiodynamics, PK, the C-QT relationship, and safety. All 24 subjects were white, the mean ± standard deviation (SD) age was 41.0 ± 11.3 years, and the mean ± SD body mass index (BMI) was 27.7 ± 3.2 kg/m^2^. Female subjects represented 75% (*n* = 18) of the total study population, but this gender imbalance was not expected to impact outcomes.

### PK analysis.

**FIG 1:**
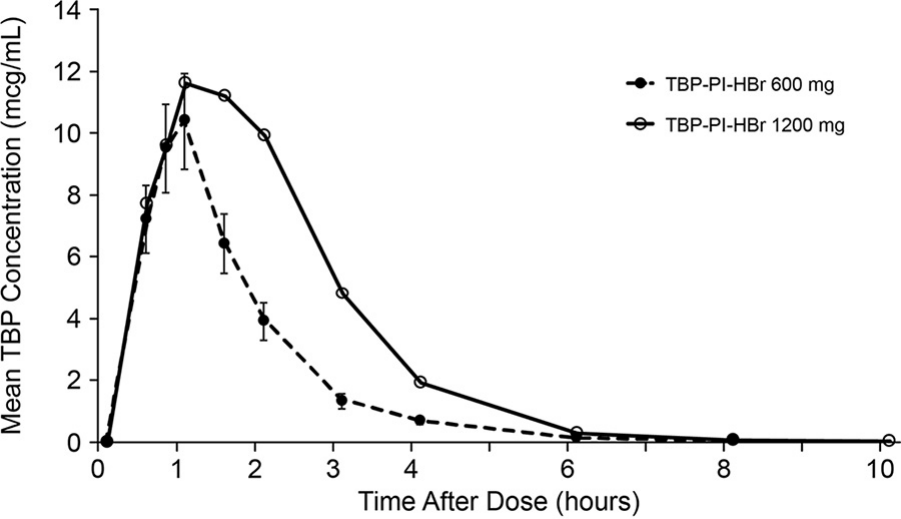
Mean plasma TBP concentrations following administration of single 600- or 1,200-mg oral doses (PK population).

Plasma TBP concentrations were measurable in all subjects by 30 min postdose for both 600- and 1,200-mg doses (Fig. 1). Following the 600-mg dose, the time at which the maximum plasma concentration (*C*_max_) occurred (*T*_max_) was 1 h for the 600-mg dose and 1.5 h for the 1,200-mg dose.

The geometric mean plasma TBP area under the concentration-time curve from time zero to infinity (AUC_0–inf_) and *C*_max_ following administration of the 600-mg dose were 16.6 μg · h/ml and 11.1 μg/ml, respectively (Table 1). The geometric mean plasma TBP *C*_max_ and AUC_0–inf_ following the 1,200-mg dose were 1.3-fold and 1.8-fold higher, respectively, than those observed following the 600-mg dose. The median TBP *T*_max_ values with the two doses were comparable, with individual subject values ranging from 0.5 to 4.0 h. The arithmetic mean half-lives of plasma TBP following the 600-mg and 1,200-mg doses were comparable and ranged from 0.9 to 1.1 h.

**TABLE 1:** Plasma TBP PK data following administration of TBP-PI-HBr in single 600-mg or 1,200-mg oral doses (PK population)

Parameter^*a*^	Data for:	
	TBP-PI-HBr at 600 mg (*n* = 24)	TBP-PI-HBr at 1,200 mg (*n* = 24)
AUC_0–_*_t_* (mean [CV]) (μg · h/ml)	16.6 (22.6)	29.0 (23.3)
AUC_0–inf_ (mean [CV]) (μg · h/ml)	16.6 (22.6)	29.0 (23.3)
AUC_%extrap_ (mean ± SD) (%)	0.10 ± 0.04	0.07 ± 0.03
*C*_max_ (mean [CV]) (μg/ml)	11.1 (28.4)	14.6 (28.0)
*T*_max_ (median [range]) (h)	1.0 (0.5–4.0)	1.5 (0.5–3.0)
*t*_1/2_ (mean ± SD) (h)	0.9 ± 0.15	1.1 ± 0.20

a The AUC and *C*_max_ values are presented as the geometric mean and geometric coefficient of variation (CV). The *t*_1/2_ is presented as the arithmetic mean ± SD.

### Cardiodynamic analysis.

The QT interval corrected by Fridericia’s method (QTcF) was determined. The overall slopes for QTcF versus RR interval were 0.0340 for placebo (drug-free) and 0.0420 for both TBP-PI-HBr doses and moxifloxacin, indicating that QTcF adequately corrected for changes in HR. In addition, mean changes in the baseline-corrected difference in HR from placebo (ddHR) of >9 beats/min (bpm) were not observed, which indicates that Fridericia’s method was an adequate method of correction.

**FIG 2:**
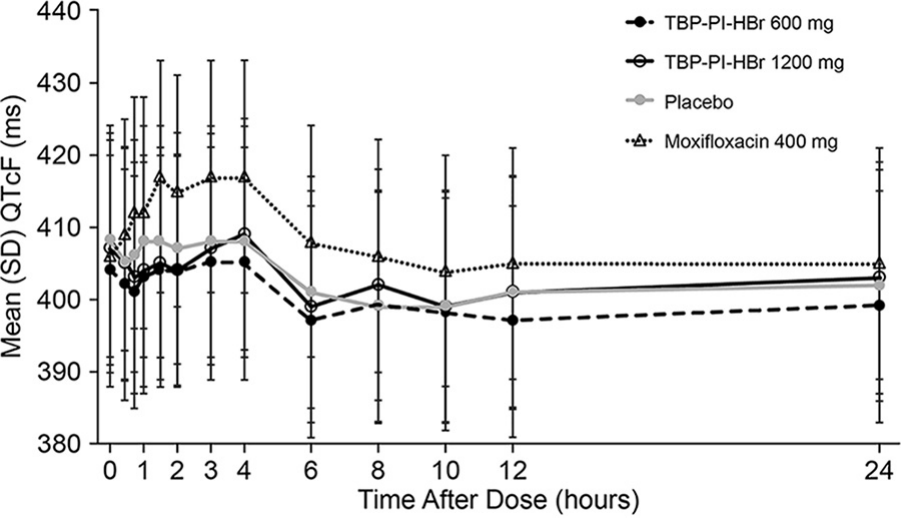
Mean ± SD QTcF versus time by treatment (cardiodynamic population).

**FIG 3:**
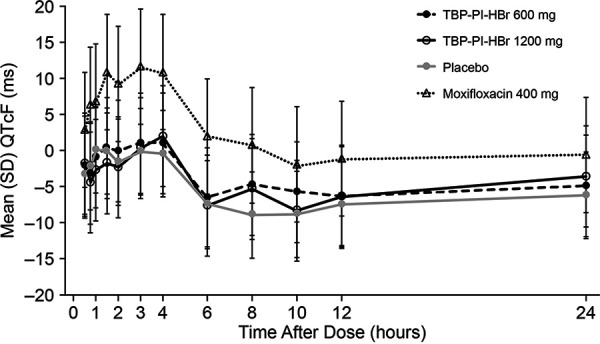
Mean ± SD dQTcF versus time by treatment (cardiodynamic population).

Mean QTcF values were <450 ms following all treatments. Mean postdose QTcF values were higher following moxifloxacin at all time points, compared to TBP-PI-HBr and placebo, with the highest mean value of 417.4 ms at hour 3. Following treatment with TBP-PI-HBr, mean QTcF values did not exceed 408.9 ms (1,200 mg of TBP-PI-HBr, hour 4), and overall mean QTcF values following active treatment were comparable to those following placebo. Mean QTcF values decreased from baseline at the majority of postdose time points following TBP-PI-HBr and placebo and increased from baseline following moxifloxacin. Through hour 4, mean changes from baseline ranged from −4.4 to +2.0 ms following both doses of TBP-PI-HBr and placebo (Fig. 2). From hours 6 through 12, decreases from baseline occurred with active treatments and placebo and were more notably negative, ranging from −4.8 ms to −8.9 ms (Fig. 3). The maximal baseline-corrected difference in QTcF from placebo (ddQTcF) for moxifloxacin was +11.9 ms (on day 1, hour 3), and the maximal ddQTcF values for TBP-PI-HBr at 600 mg and 1,200 mg were +4.1 ms and +3.6 ms, respectively, on day 1, hour 8. Overall ddQTcF values for active treatments ranged from −2.8 ms to +4.1 ms.

TBP-PI-HBr had no clinically significant adverse effects on HR or electrocardiogram (ECG) parameters, including PR interval, QRS interval, QT interval, RR interval, HR-corrected QT interval (QTcF and QT interval corrected by Bazett's method [QTcB]), and ECG morphology.

### Categorical analysis.

One subject experienced postdose triplicate-average QTcF values of >450 ms to ≤480 ms following both TBP-PI-HBr at 1,200 mg (451 ms at hour 1.5) and moxifloxacin at 400 mg (hours 0.50, 0.75, and 24.0). This subject also had QTcB values of >450 ms to ≤480 ms at multiple time points following all four treatments, including placebo, with a maximum value of 460.7 ms. One subject experienced a QTcB value of >450 ms to ≤480 ms following moxifloxacin (hour 10), with a value of 452.3 ms. No subject had a ddQTcF of ≥30 ms following any treatment. Four subjects experienced a change from baseline of ≥30 ms to ≤60 ms in QTcB values, including 1 subject with both TBP-PI-HBr at 1,200 mg and moxifloxacin at 400 mg and 3 subjects with moxifloxacin at 400 mg only. The maximum ddQTcB value reported for these subjects was 43.5 ms.

### C-QT.

**FIG 4:**
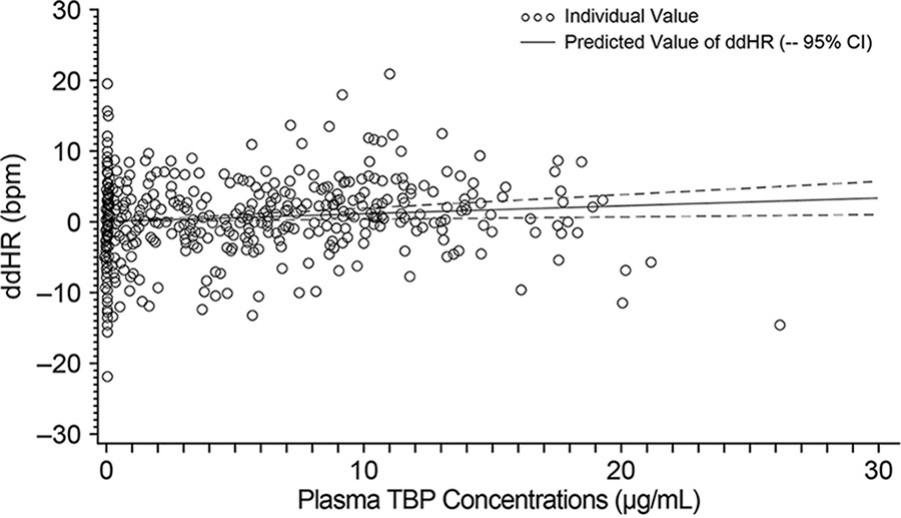
ddHR versus time-matched plasma TBP concentration (C-QT population).

For the analysis of ddHR versus time-matched plasma TBP concentrations, the slope and intercept based on the model were 0.11 (90% confidence interval [CI], 0.02 to 0.20) and 0.05 (90% CI, −0.53 to 0.63), respectively (Fig. 4). The model-predicted slope suggests that ddHR was not importantly affected by plasma TBP concentrations, supporting the use of QTcF as an appropriate correction method.

A delay of more than 1 h was observed between the time of the largest mean difference in QTcF change from baseline between treatment and placebo and *T*_max_ for TBP-PI-HBr at 600 mg and 1,200 mg. However, mean ddQTcF values for both doses at each time point were <5 ms. Therefore, although hysteresis loop plots contained counterclockwise elements, no meaningful PK hysteresis effect was observed between plasma TBP concentrations and ddQTcF versus time for TBP-PI-HBr at 600 mg and 1,200 mg.

**FIG 5:**
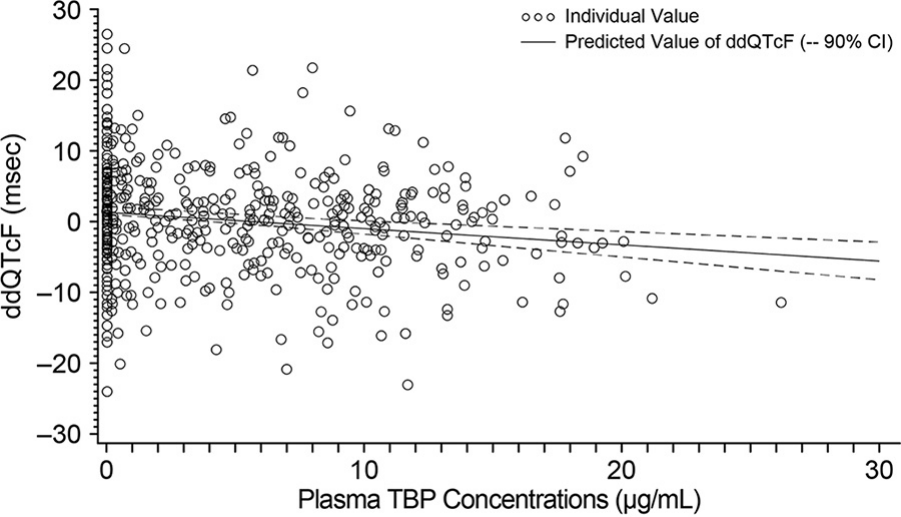
ddQTcF versus time-matched plasma TBP concentration (C-QT population).

**FIG 6:**
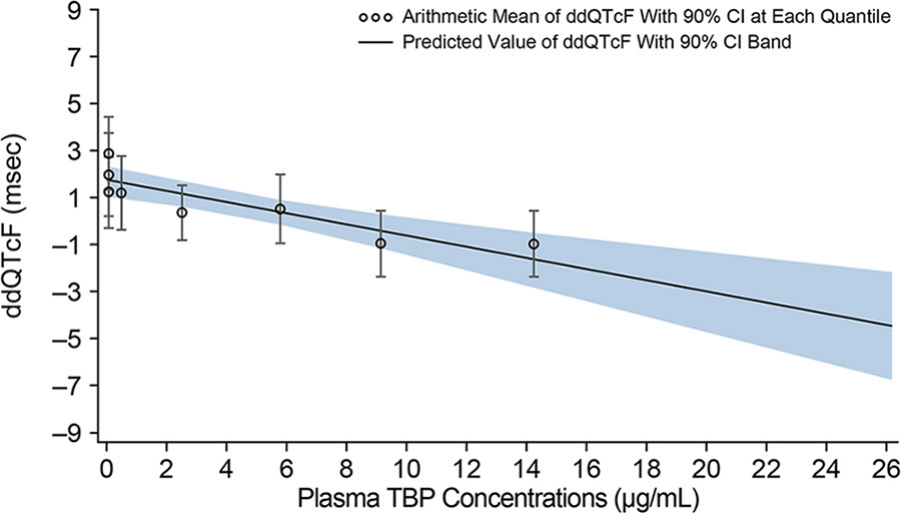
ddQTcF versus plasma TBP concentration (C-QT population).

For baseline-adjusted QTcF (dQTcF) versus plasma TBP concentrations, the slope and intercept based on the model were 0.1860 (95% CI, 0.0807 to 0.2913) and −3.8210 (95% CI, −4.5041 to −3.1378), respectively. The outliers at the zero TBP concentration level modified the slope, and the CI of the fitted line failed to capture most data at lower plasma concentrations. The relationship between ddQTcF and plasma TBP concentrations was examined to exclude the potential placebo effect at the zero plasma TBP concentration level. The relationship between ddQTcF and plasma TBP concentrations is shown in Fig. 5. The slope and intercept based on the model were −0.2379 (90% CI, −0.3396 to −0.1362) and 1.7493 (90% CI, 1.0894 to 2.4093), respectively. The plot of ddQTcF versus plasma TBP concentrations showed a better model fit and was deemed a more appropriate model for the analysis (Fig. 6).

The predicted maximum values for ddQTcF at the geometric mean *C*_max_ for each TBP-PI-HBr dose (11.1 and 14.6 μg/ml for 600 mg and 1,200 mg, respectively) were negative (600 mg: −0.9 [90% CI, −1.79 to −0.01]; 1,200 mg: −1.7 [90% CI, −2.93 to −0.53]). Therefore, no QTc prolongation (>10 ms) was detected at the peak concentration for either the 600-mg dose or the 1,200-mg dose of TBP-PI-HBr. Assay sensitivity was established with a lower limit of the two-sided 90% CI of predicted maximum ddQTcF at *C*_max_ for moxifloxacin of >5 ms (16).

### Safety and tolerability.

Overall, 8 subjects (33%) reported 24 treatment-emergent adverse events (TEAEs) (Table 2). No adverse events (AEs) were reported with placebo. The most common AE was headache. Twelve AEs were considered probably or possibly related to the study drug. All drug-related AEs were reported following TBP-PI-HBr at 1,200 mg or moxifloxacin. All AEs were mild (18 events) or moderate (5 events) in severity, with the exception of 1 severe event of syncope following 1,200 mg of TBP-PI-HBr. The severe syncope occurred 18.3 h after the administration of TBP-PI-HBr, was possibly affected by poor food intake, was not associated with ECG abnormalities, and was considered possibly related to the study drug. All AEs resolved by study completion. No deaths, serious TEAEs, study withdrawals, or premature discontinuations of study drug due to TEAEs were reported.

**TABLE 2:** TEAEs occurring in at least 1 subject with TBP-PI-HBr

TEAE	No. (%) of subjects who received:		
	TBP-PI-HBr at 600 mg (*n* = 24)	TBP-PI-HBr at 1,200 mg (*n* = 24)	Moxifloxacin (*n* = 24)
At least 1 TEAE	3 (13)	4 (17)	4 (17)
Photophobia	1 (4)	0 (0)	0 (0)
Diarrhea	0 (0)	1 (4)	1 (4)
Feeling cold	0 (0)	1 (4)	0 (0)
Urinary tract infection	1 (4)	0 (0)	0 (0)
Increased HR	1 (4)	0 (0)	0 (0)
Dizziness	0 (0)	1 (4)	1 (4)
Headache	2 (8)	1 (4)	1 (4)
Syncope	0 (0)	1 (4)	0 (0)
Dyspnea	0 (0)	1 (4)	0 (0)
Nasal congestion	0 (0)	1 (4)	0 (0)
Sneezing	0 (0)	1 (4)	0 (0)
Pruritus	0 (0)	1 (4)	0 (0)

No clinically significant changes in laboratory results were observed, and vital signs remained within normal limits throughout the study, with minimal changes from baseline. Mean safety ECG parameters remained within normal limits, with minimal changes from baseline, for all treatments. No AEs were observed to be related to ECG findings, and no abnormal findings were considered clinically significant.

## DISCUSSION

This study demonstrated that, after single doses of TBP-PI-HBr, there were no clinically significant adverse effects on HR or ECG parameters, including PR interval, QRS interval, QT interval, RR interval, HR-corrected QT interval (QTcF and QTcB), and ECG morphology. No QTc prolongation was detected following administration of 600 mg or 1,200 mg TBP-PI-HBr, and the model-predicted ddQTcF at the geometric mean *C*_max_ for plasma TBP had negative predicted values (90% CIs) for both the therapeutic (600-mg) and supratherapeutic (1,200-mg) doses of TBP-PI-HBr. Assay sensitivity was established using the same model with moxifloxacin, which is an established positive control for QTc studies (16). The PK profile of TBP was characterized by a dose-dependent increase in exposure with 600-mg and 1,200-mg doses of TBP-PI-HBr. At a 1,200-mg dose, TBP AUC_0–inf_ and *C*_max_ were 1.8- and 1.3-fold higher, respectively, than those observed with the 600-mg dose. No new safety signals with TBP-PI-HBr were detected in this study, compared with an earlier phase 1 study with healthy subjects.

In nonclinical studies, *in vitro* TBP had no effect on the human ether-a-go-go-related gene (hERG) channel under serum-free conditions up to a maximum concentration of 115 μg/ml (17). No significant effect on cardiovascular functioning, including ECGs, was observed in monkeys with oral administration of up to 300 mg/kg TBP-PI (Spero Therapeutics, data on file). In dogs, mild and transient decreases in HR and blood pressure were noted with a TBP-PI dose of 100 mg/kg, with no effects at 10 or 30 mg/kg (Spero Therapeutics, data on file).

Clinical development of a novel compound such as TBP-PI-HBr, the first oral carbapenem to be developed for U.S. approval, includes an assessment of effects on the QTc as part of clinical pharmacology studies. The design of this study was consistent with FDA guidance and incorporated therapeutic and supratherapeutic doses of TBP-PI-HBr, as well as a 400-mg dose of moxifloxacin to confirm the assay sensitivity (6). Rather than a thorough QT interval study as previously required, this study used the C-QT relationship model to characterize the effect of drug concentrations on QT interval prolongation (18). For safety purposes, the study design used here included a sentinel group in which 1 subject each received TBP-PI-HBr, moxifloxacin, and placebo in period 1 to assess safety prior to the completion of dosing in all subjects.

A 600-mg dose of TBP-PI-HBr administered orally every 8 h is the therapeutic dose used in a phase 3 study to treat patients with complicated urinary tract infections and acute pyelonephritis (19). A supratherapeutic dose of 1,200 mg TBP-PI-HBr has not previously been administered to healthy adults, but a single 900-mg dose and a multiple-dose regimen of 600 mg every 8 h for up to 14 days have been administered to healthy subjects and were well tolerated (15). Because of the short half-life (<1 h) of TBP in humans, no evidence of accumulation was observed with TBP-PI-HBr at 600 mg every 8 h for 14 days (15). Further, TBP has no active metabolites that could contribute to adverse effects on the QT interval. In the phase 1 single-dose/multiple-ascending-dose study, TBP-PI-HBr at 300 mg and 600 mg administered every 8 h produced *C*_max_ values ranging from 6.5 to 7.8 μg/ml and from 13.4 to 15.1 μg/ml, respectively (15). The supratherapeutic 1,200-mg dose used in this study was expected to produce a *C*_max_ that was 1.3-fold higher and an AUC_0–inf_ that was 1.8-fold higher than those observed with the 600-mg dose. Nine of 24 subjects had *C*_max_ values greater than the target (at least 1.5-fold greater) with no effect on the QTc. The *C*_max_ and AUC with the 1,200-mg dose used in this study were expected to be lower than the no-observed-adverse-effect levels in rats and monkeys (Spero Therapeutics, data on file).

In summary, based on the results of this study, TBP-PI-HBr is unlikely to have a clinically significant effect on the QT interval at therapeutic or supratherapeutic doses.

## MATERIALS AND METHODS

### Ethics statement.

The study was conducted in accordance with the U.S. Code of Federal Regulations and the ethical principles of the Declaration of Helsinki, good clinical practices, and the International Council for Harmonsation guidelines. The study protocol and all amendments were reviewed by the institutional review board for the study center (Advarra Institutional Review Board, Columbia, MD). Informed consent was obtained from each subject in writing before any study-related procedure, including randomization, occurred.

### Study design.

This was a randomized, double-blind, placebo- and active-controlled, four-way crossover study using a Latin squares design that was balanced for carryover effects. The active control, moxifloxacin, was administered open label. Eligible subjects were randomly assigned to receive a single dose of one of four study treatments, separated by a 7-day washout period. On day 1 of period 1, subjects were randomly assigned to 1 of 12 treatment sequences. In period 1 only, 4 subjects were dosed 24 h prior to the remaining 20 subjects. Each of the 4 subjects from the sentinel group received a different treatment. On day 1 of each period, subjects were randomly assigned to receive a single oral dose of TBP-PI-HBr at 600 mg (two 300-mg tablets) and two matching placebo tablets, a supratherapeutic dose of TBP-PI-HBr at 1,200 mg (four 300-mg tablets), placebo, or moxifloxacin at 400 mg. All study drugs were administered with 240 ml of water at time zero of day 1 of each treatment period, after an overnight fast.

### Subject selection.

Men or women with an age of 18 to 65 years and a BMI of ≥18.0 and ≤32.0 kg/m^2^ at screening were eligible if they were medically healthy with no clinically significant abnormalities in the medical history, physical examination, laboratory testing, vital signs, or ECG. Subjects were nonsmokers with no use of nicotine-containing products for ≥3 months. Subjects had to have a normal sinus rhythm (HR of 50 to 100 bpm), QTcF of ≤450 ms, QRS interval of <100 ms, and PR interval of <200 ms.

Subjects were excluded for a history of ventricular preexcitation syndrome (Wolff-Parkinson-White syndrome); arrhythmia or a history of arrhythmia requiring medical intervention; risk factors for torsade de pointes (e.g., heart failure, cardiomyopathy, or a family history of long QT syndrome); or sick sinus syndrome, second- or third-degree atrioventricular block, myocardial infarction, pulmonary congestion, symptomatic or significant cardiac arrhythmia, prolonged QTcF, or conduction abnormalities. Subjects also were excluded for seated blood pressure of >140/90 mm Hg or <90/40 mm Hg, seated HR of <40 or >99 bpm, or estimated creatinine clearance of <80 ml/min at screening. Women of childbearing potential had to use an effective method of birth control from ≥3 months prior to the first dose to 28 days after the study and had to have a negative pregnancy test result at screening and on the day prior to dosing.

### Study assessments.

At screening, all subjects underwent a comprehensive physical examination and provided a medical history; vital signs (blood pressure and HR), safety 12-lead ECG, and routine laboratory test (hematology, chemistry, and urinalysis) results were obtained. Serology tests, urine drug screening, and pregnancy tests (female subjects only) were performed.

A single (triplicate predose in period 1 only) 12-lead ECG was performed within 2 h prior to dosing on day 1 and then 1, 2, 8, and 24 h after dosing during resting in the supine position in a quiet environment. Holter monitors were used to collect continuous 12-lead ECG data for cardiodynamic monitoring. Triplicate 10-s, 12-lead ECG recordings were extracted from Holter monitor data within a 5-min time window but prior to PK blood sample collection. Timing and recording techniques for ECGs were standardized for all subjects. Subjects were required to lie quietly in a supine position with minimal movement and minimal exposure to noise and other environmental stimuli for at least 10 min before and 5 min during the ECG extraction, to allow quality ECG extraction. If targeted ECG time points were artifactual or of poor quality, then analyzable 10-s ECGs were extracted as close as possible to the targeted time points. All ECG tracings were manually reviewed by the investigator in a blinded manner.

Blood samples were obtained predose and 0.5, 0.75, 1, 1.5, 2, 3, 4, 6, 8, 10, 12, and 24 h after the dose to measure TBP and moxifloxacin plasma levels. Plasma concentrations less than the lower limit of quantification were reported as zero. Whole blood samples were assayed for TBP using a validated liquid chromatography-tandem mass spectrometry (LC-MS/MS) method (Charles River Laboratories, Shrewsbury, MA). TBP blood concentrations were converted to plasma concentrations before the PK analysis, as follows: plasma concentration = reported blood concentration × 3.6 mcg/ml, where 3.6 represents the product of a 1/plasmatocrit value of 1.8 (using an average plasmatocrit value of 0.55) and an isopropyl alcohol (IPA) dilution factor of 2. IPA was added as a stabilizer during blood sample collections to prevent conversion of TBP-PI-HBr to TBP after sample collection. Plasma concentrations of moxifloxacin were determined using a validated LC-MS/MS method (WuXi AppTec, Laboratory Testing Division, Plainsboro, NJ).

Noncompartmental PK parameters included *C*_max_, *T*_max_, apparent first-order terminal elimination rate constant (*k*_el_) calculated from the plasma concentration-time curve, terminal elimination half-life calculated as 0.693/*k*_el_, AUC_0–inf_, AUC from time zero to the last non-zero value (AUC_0–last_), and percentage of AUC_0–inf_ extrapolated (AUC_%extrap_), represented as ([1 − AUC_0–last_/AUC_0–inf_] · 100) and calculated by the linear-up/logarithmic-down trapezoidal method. PK parameters were determined using Phoenix WinNonlin (version 8.0), and statistical comparisons used SAS version 9.4.

Safety was assessed from vital signs (blood pressure, HR, respiratory rate, and body temperature), physical examination and clinical laboratory test (serum chemistry, hematology, and urinalysis) results, safety ECGs, and AEs.

### Statistical analysis.

Based on published literature on thorough QT interval studies (17, 20, 21), 8 or 9 subjects would provide >80% power to detect a significant drug-induced QTc prolongation. Since this study did not use a supratherapeutic dose of moxifloxacin (positive control), a greater number of subjects receiving only a single dose of moxifloxacin was utilized (i.e., *n* = 20). Thus, an *n* value of 24 subjects was chosen for the study to ensure that data from 20 subjects with evaluable observations for each of moxifloxacin and placebo could be obtained. Collectively, based on literature and regulatory discussions for moxifloxacin, this sample size was deemed appropriate to detect a drug-induced QTc prolongation by TBP of greater than 5 ms and accounts for potential dropouts.

The predose dQTcF was defined at each postdose time point as the difference between the QTcF value for that time point on the treatment day minus the predose QTcF for the corresponding time point. The ddQTcF was calculated for each active treatment at each postdose time point as the dQTcF for that treatment minus the corresponding dQTcF for the placebo.

The potential relationship between TBP plasma concentrations and dQTc (C-QT) was explored using a linear mixed-effect model by regressing dQTc on time-matched TBP concentrations using SAS PROC MIXED and data across all dose levels.

After confirming the most adequate method of QT interval correction, hysteresis was assessed by plotting mean drug concentrations and mean ddQTc values over time by dose levels. To detect hysteresis, individual ddQTc values from subjects who received TBP were computed as dQTc minus the time-matched dQTc when the subject received placebo. The time of the largest mean ddQTc (*U*_max_) was determined. If the mean ddQTc exceeded 5 ms at more than three time points, the time difference between *U*_max_ and *T*_max_ exceeded 1 h, and the one-sided one-sample Wilcoxon test for the difference between ddQTc at *T*_max_ and at *U*_max_ was significant at the 1% level, it was concluded that hysteresis existed. In such a case, a PK model with an additional effect compartment replaced this model. In addition, individual subject hysteresis loops were presented if hysteresis was detected. The relationship between dQTcF and moxifloxacin plasma concentrations (C-QT relationship) also was assessed using a linear mixed-effect model.

A mixed-effect analysis of covariance (ANCOVA) for a four-period crossover design was used to analyze dQTc at each time point postbaseline. The model included fixed effects for sequence, period, treatment, time, and the interaction terms for time and treatment and for time and period. The covariate was baseline value, and the repeated variable was the time point at which the QTc was measured. Subject nested in sequence was included in the mixed model as a random effect, and an appropriate within-subject variance-covariance structure was specified. Mean ddQTc and two-sided 90% CI for each time point were derived. For the purpose of assessing drug-induced changes in HR, ddHR with 90% CI for each time point was generated.

Categorical analyses determined the proportions of subjects who met the following criteria: QT interval and QTc of ≤450, >450, >480, and >500 ms; QTc increase from baseline of <30, ≥30, and ≥60 ms; HR of <50, <40, <30, and >900 bpm; and change from baseline of <30% and ≥30% and of <25% and ≥25% for PR and QRS intervals.

The safety population included all subjects who were randomly assigned and received at least one dose of study drug. The PK population was all subjects with evaluable concentration-time profiles following administration of study drug and no major protocol violations that impacted the PK analysis. Subjects who did not complete the PK sampling as scheduled but had sufficient data points to construct a concentration-time profile were considered PK evaluable. The cardiodynamic population included all subjects who received TBP-PI-HBr, moxifloxacin, or placebo and had valid day 1 QT/QTc interval measurements (i.e., predose and at least one postdose measurement). The C-QT population was all subjects who received at least one dose of TBP-PI-HBr, moxifloxacin, or placebo, had at least one valid day 1 QT/QTc interval measurement predose and postdose, and had a time-matched QTc/PK assessment included in the C-QT relationship analysis.
